# Digital communication between mothers and community health workers to support neonatal health (CHV-NEO): study protocol for a randomized controlled trial

**DOI:** 10.1186/s13063-024-08501-2

**Published:** 2024-10-04

**Authors:** Keshet Ronen, Lincoln C. Pothan, Violet Apondi, Felix A. Otieno, Daniel Mwakanema, Felix O. Otieno, Lusi Osborn, Julia C. Dettinger, Priyanka Shrestha, Helena Manguerra, Ferdinand Mukumbang, Millicent Masinde, Evelyn Waweru, Mercy Amulele, Christine Were, Beatrice Wasunna, Grace John-Stewart, Bryan Weiner, Arianna Rubin Means, Barbra A. Richardson, Anna B. Hedstrom, Jennifer A. Unger, John Kinuthia

**Affiliations:** 1 Department of Global Health, University of Washington, Seattle, WA USA; 2 Department of Research and Programs, Kenyatta National Hospital, Nairobi, Kenya; 3 Department of Obstetrics and Gynecology, Kenyatta National Hospital, Nairobi, Kenya; 4 Medic, Nairobi, Kenya; 5 Department of Pediatrics, University of Washington, Seattle, WA USA; 6 Department of Medicine, University of Washington, Seattle, WA USA; 7 Department of Epidemiology, University of Washington, Seattle, WA USA; 8 Department of Obstetrics and Gynecology, Brown University, Women and Infants Hospital, Providence, RI USA; 9 Department of Biostatistics, University of Washington, Seattle, WA USA; 10 Blantyre, Malawi

**Keywords:** SMS, Essential newborn care, Neonatal mortality, Kenya, Community health worker, Implementation Science, mHealth

## Abstract

**Background:**

Provision of essential newborn care at home, rapid identification of illness, and care-seeking by caregivers can prevent neonatal mortality. Mobile technology can connect caregivers with information and healthcare worker advice more rapidly and frequently than healthcare visits. Community health workers (CHWs) are well-suited to deliver such interventions. We developed an interactive short message service (SMS) intervention for neonatal health in Kenya, named CHV-NEO. CHV-NEO sends automated, theory-based, actionable, messages throughout the peripartum period that guide mothers to evaluate maternal and neonatal danger signs and facilitate real-time dialogue with a CHW via SMS. We integrated this intervention into Kenya’s national electronic community health information system (eCHIS), which is currently used at scale to support CHW workflow.

**Methods:**

The effect of CHV-NEO on clinical and implementation outcomes will be evaluated through a non-blinded cluster randomized controlled trial. Twenty sites across Kisumu County in Western Kenya were randomized 1:1 to provide either the national eCHIS with integrated CHV-NEO messaging (intervention) or standard of care using eCHIS without CHV-NEO (control). We will compare neonatal mortality between arms based on abstracted eCHIS data from 7200 pregnant women. Secondary outcomes include self-reported provision of essential newborn care (appropriate cord care, thermal care, and timely initiation of breastfeeding), knowledge of neonatal danger signs, and care-seeking for neonatal illness, compared between arms based on questionnaires with a subgroup of 2000 women attending study visits at enrollment in pregnancy and 6 weeks postpartum. We will also determine CHV-NEO’s effect on CHW workflows and evaluate determinants of intervention acceptability, adoption, and fidelity of use through questionnaires, individual interviews, and messaging data.

**Discussion:**

We hypothesize that the CHV-NEO direct-to-client communication strategy can be successfully integrated within existing CHW workflows and infrastructure, improve the provision of at-home essential newborn care, increase timely referral of neonatal illness to facilities, and reduce neonatal mortality. The intervention’s integration into the national eCHIS tool will facilitate rapid scale-up if it is clinically effective and successfully implemented.

**Trial registration:**

ClinicalTrials.gov, NCT05187897. The CHV-NEO study was registered on January 12, 2022.

## Administrative information

Note: the numbers in curly brackets in this protocol refer to SPIRIT checklist item numbers. The order of the items has been modified to group similar items (see http://www.equator-network.org/reporting-guidelines/spirit-2013-statement-defining-standard-protocol-items-for-clinical-trials/).

**Table:** 

Title {1}	Digital communication between mothers and community health workers to support neonatal health (CHV-NEO): Study protocol for a randomized controlled trial
Trial registration {2a and 2b}.	ClinicalTrials.gov Identifier: NCT05187897
Protocol version {3}	28 November 2023, V.5
Funding {4}	**Funding type:** Grant **Name of Funding agency:** NIH/NICHD **Grant Number:** 1 R01 HD103581
Author details {5a}	Department of Global Health, University of Washington, Seattle, WA, USA Department of Research & Programs, Kenyatta National Hospital, Nairobi, Kenya Department of Obstetrics and Gynecology, Kenyatta National Hospital, Nairobi, Kenya Medic, Nairobi, Kenya Department of Pediatrics, University of Washington, Seattle, WA, USA Department of Medicine, University of Washington, Seattle, WA, USA Department of Epidemiology, University of Washington, Seattle, WA, USA Department of Obstetrics and Gynecology, Brown University, Women and Infants Hospital, Providence, RI, USA Department of Biostatistics, University of Washington, Seattle, WA, USA
Name and contact information for the trial sponsor {5b}	University of Washington osp@uw.edu
Role of sponsor {5c}	The funder (National Institutes of Health) and sponsor (University of Washington) had no role in the design of this study. They will not have any role during its execution, analyses, interpretation of the data, or decision to submit results.

## Introduction

### Background and rationale {6a}

Despite recent reductions in under-five mortality, neonatal mortality (i.e., mortality in the first 28 days of life) remains high in low- and middle-income countries (LMICs). For neonates who come home after facility delivery, subsequent deaths are the result of complications of preterm delivery and neonatal infections [1]. For each of these causes of death, evidence-based prevention and management strategies exist [2, 3]. Early initiation of breastfeeding (in the first hour of life) and exclusive breastfeeding (EBF) are independently associated with lower neonatal mortality [4]. Clean cord care can prevent cord infections, sepsis, and neonatal mortality [5, 6]. Thermal care, including skin-to-skin contact with the mother, drying and wrapping immediately after birth, and delaying bathing for at least 24 h, prevents hypothermia and associated morbidity and mortality, particularly in preterm babies [7]. The World Health Organization (WHO) recommends home implementation of thermal care [8]. Despite evidence for the efficacy of these interventions, coverage remains low in many African settings [9–12]. Caregiver provision of essential newborn care (ENC) at home requires accurate information, motivation, behavioral skills, and an enabling environment [13].

When neonates develop illness, timely and appropriate care-seeking is essential for survival. Many newborns continue to die at home without health care being sought because of delays that prevent postpartum women and neonates from accessing the care they need. The “three delays model,” originally developed to understand maternal deaths [14], has been adapted to assess missed opportunities leading to neonatal deaths. The model identifies delays in (1) identifying illness and deciding to seek care, (2) reaching the health facility, and (3) receiving quality care once a facility is reached. Prior studies suggest delays recognizing illness and deciding to seek care (delay 1) account for up to 80% of neonatal and child deaths [15–18].

Community health workers (CHWs) are a large cadre of community-based lay health workers that conduct home visits in pregnancy and the neonatal period, provide preventative care, health education, and referrals to seek care when needed. Home visits by CHWs supplement facility-based care and promote family contact with the health system at crucial times. A number of studies in LMIC settings demonstrate that home-based neonatal care by trained CHWs can significantly increase ENC [19–21], improve the identification of neonatal illness [20–22], facilitate referrals, and ultimately prevent neonatal mortality [23, 24]. Although the provision of home-based care by CHWs has demonstrated a positive impact, it is limited by the fact that visits are intermittent. Despite recommendations of one to four home visits in the early neonatal period, CHWs have difficulty meeting their visit goals due to access challenges in large catchment areas and lack of knowledge, supervision, and motivation [21, 25, 26]. In addition, CHWs are not “on-call” and intermittent visits may miss critical periods when neonates become ill and quickly decline.

Mobile health (mHealth) interventions are increasingly used in a variety of contexts to supplement healthcare provider encounters with more frequent, on-demand, and anticipatory guidance. In Kenya, for example, the National Community Health Digitization Strategy 2020–2025 launched an effort to digitize community health services across the country so that CHWs use a tablet-based electronic community health information system (eCHIS) to manage service delivery and data capture [27]. According to the Communications Authority of Kenya, there are now 46 million phone subscriptions in Kenya, for a national population of 43 million [28]. Short messaging service (SMS) is an accessible modality that can be used to educate, provide reminders, and improve communication between healthcare workers and patients [29–32]. There is also evidence that mHealth interventions improve antenatal care (ANC) and PNC attendance, skilled delivery uptake, and EBF [33–37]. Few studies have evaluated neonatal outcomes [38].

We previously developed an interactive, semi-automated SMS intervention that connects perinatal clients with nurses, named Mobile WACh [39]. Mobile WACh message content has been adapted for a variety of maternal and child health outcomes and shown in randomized studies to increase the duration of EBF and postpartum uptake of contraception [37, 40]. An ongoing trial evaluates the efficacy of the approach in improving neonatal outcomes [41]. However, no studies have evaluated the integration of Mobile WACh into routine service delivery or tested intervention delivery by CHWs.

### Objectives {7}

CHV-NEO integrates Mobile WACh into Kenya’s national eCHIS, resulting in an interactive SMS intervention that connects perinatal clients with CHWs. Our objective is to pragmatically evaluate: (1) the effect of CHV-NEO on neonatal mortality, ENC provision, and care-seeking; (2) the effect of CHV-NEO on CHW service delivery outcomes; and (3) levels and determinants of CHV-NEO implementation outcomes. We hypothesize that mother–infant dyads at facilities randomized to CHV-NEO will demonstrate lower neonatal mortality, higher provision of ENC, and higher facility care-seeking compared with standard of care and that frequency of CHW routine home visits will not differ between arms.

### Trial design {8}

This study is a non-blinded cluster-randomized controlled superiority trial comparing two parallel arms (intervention vs. control). The study intervention is delivered by CHWs who are employed by Kisumu County and whose work takes place at the household and community level. Each CHW is assigned by the county to a community area for which they are responsible. Each CHW is affiliated with and supervised through a nearby assigned healthcare facility. The unit of randomization in our trial is the healthcare facility and the associated CHWs and community areas, referred to here as “facility clusters”. Twenty clusters were randomized to the intervention (eCHIS with integrated SMS, referred to as CHV-NEO) or the control (routine eCHIS without SMS) in a 1:1 allocation ratio (10 control clusters, 10 intervention clusters). All CHWs linked to each facility cluster provide the assigned intervention to their community-based clients.

## Methods: participants, interventions, and outcomes

### Study setting {9}

This study takes place in community areas linked to 20 government healthcare facilities in Kisumu County, Kenya. Kenya has approximately 30,000 neonatal deaths each year [42], with a neonatal mortality rate of 21.0 per 1000 live births in Kisumu [43]; this is substantially higher than Sustainable Development Goal 3 of ≤ 12 neonatal deaths per 1000 live births by 2030 [44]. Facilities were selected to include a diversity of characteristics, including rural and urban location, a range of facility levels from health centers (level 3) to county hospitals (level 5), and patient volumes of 100–1000 monthly antenatal care visits.

### Eligibility criteria {10}

The study enrolls four groups of participants to evaluate different outcomes: pregnant women for a mortality cohort, pregnant women for an in-depth data collection cohort, CHWs, and CHW supervisors.

#### Pregnant women—mortality cohort

**Fig. 1:**
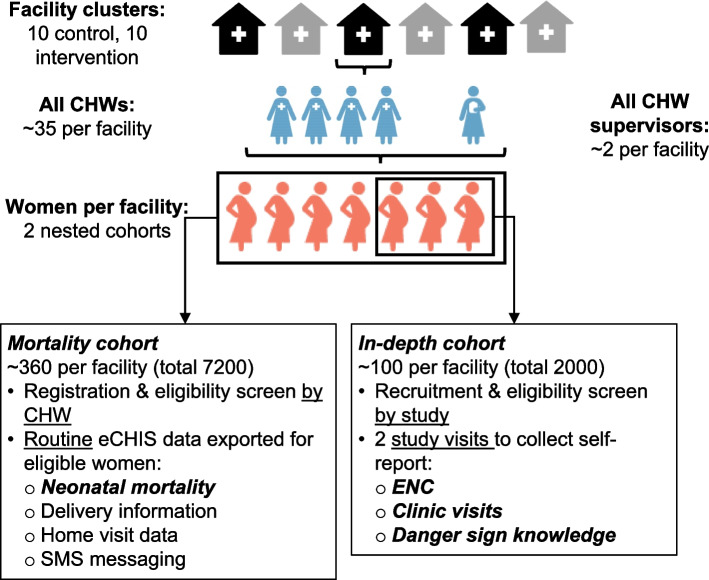
Study design overview

Outcomes are ascertained from two nested cohorts of pregnant women (Fig. 1). The primary clinical effectiveness outcome (neonatal mortality) is ascertained using abstracted routine data from the eCHIS platform in all study sites. Inclusion criteria for these women are being registered as pregnant in eCHIS at gestational age ≤ 36 weeks by a CHW linked to a study facility.

#### Pregnant women—in-depth cohort

Secondary clinical outcomes and implementation outcomes are ascertained among a nested cohort of women recruited by the study team when attending ANC visits at study facilities. Eligible participants for this cohort are pregnant (26–36 weeks gestation); registered as pregnant in eCHIS by a CHW linked to the study facility; age ≥ 14; receiving ANC at a study facility; able to read and respond to text messages in English, Kiswahili, or Luo or have someone in the household who can assist; have daily access to a mobile phone (own or shared) on Safaricom or Airtel networks; and plan to be in the area for at least 3 months postpartum. Participants who are enrolled in another research study or have previously been enrolled in CHV-NEO in a prior pregnancy are excluded.

#### CHWs and supervisors

Service delivery and implementation outcomes are ascertained from CHWs and CHW supervisors. CHWs and supervisors age ≥ 18 who are working in communities linked to the study facilities are eligible to participate in the study.

### Who will take informed consent? {26a}

#### In-depth pregnant cohort, CHWs, and supervisors

Following initial recruitment, a verbal explanation of the study is provided by study staff and verbal consent is sought to conduct eligibility screening. If the participant is eligible, written consent is obtained electronically by study staff at the time of recruitment, using REDCap [45]. Participants are informed that participation is completely voluntary and that they are free to decline participation without losing their regular medical care or their employment. Pregnant women age < 18 are considered emancipated minors under Kenyan regulations so a waiver of parental permission was obtained for these participants to allow them to provide consent independently.

#### Mortality cohort

Neonatal mortality is ascertained using abstracted, deidentified data from the eCHIS database for clients of CHWs affiliated with study facilities. These participants are not enrolled in the study as human subjects and do not provide consent for study activities.

#### Additional consent provisions for collection and use of participant data and biological specimens {26b}

N/a. The study is not collecting biological specimens.

## Interventions

### Explanation for the choice of comparators {6b}

Randomization is at the level of the healthcare facility. A cluster-randomized design is used in which entire facilities are randomized because the intervention impacts CHW workflow and therefore affects all households under their care rather than individual clients. The comparison control treatment is the standard of care version of eCHIS which is currently being used by CHWs across Kenya. eCHIS guides CHWs through completing home visits, with a range of workflows including maternal health, child health, nutrition, malaria, and water sanitation and hygiene [46]. This version of eCHIS has no SMS communication between CHWs and clients. In both study arms, antenatal and maternal and child health clinical services continue to be provided through MOH clinics with minimal interactions with study personnel.

### Intervention description {11a}

Intervention sites utilize a modified version of eCHIS which has SMS messaging incorporated as an additional module (CHV-NEO).

#### SMS curriculum

**Fig. 2:**
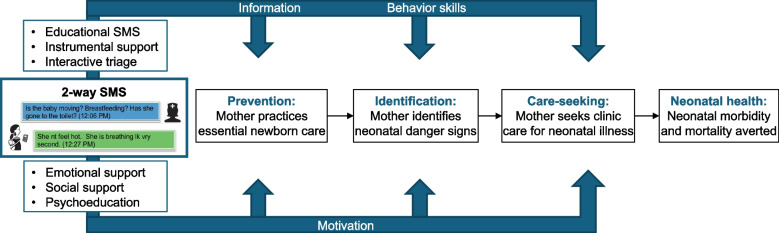
Intervention conceptual framework

The CHV-NEO SMS intervention is based on the content of the Mobile WACh NEO intervention [41], with adaptations for this study guided by input from perinatal clients, nurses, CHWs, and CHW supervisors in formative interviews and focus groups. The intervention is based on the Information, Motivation, Behavioral skills model of behavioral change [47], with the goal of improving the provision of preventative ENC, identification of illness, and care-seeking if needed (Fig. 2). Clients receive a curriculum of pre-composed SMS messages automatically sent on a schedule starting at 28 weeks gestation and extending until 6 weeks postpartum. Clients can send an SMS at any time and their message will be read and manually responded to by the CHW who routinely conducts their home visits. Automated messages are sent in English, Luo, or Kiswahili depending on the participant’s preference. Sending and receiving SMS is at no cost to the participant or the CHW.

**Fig. 3:**
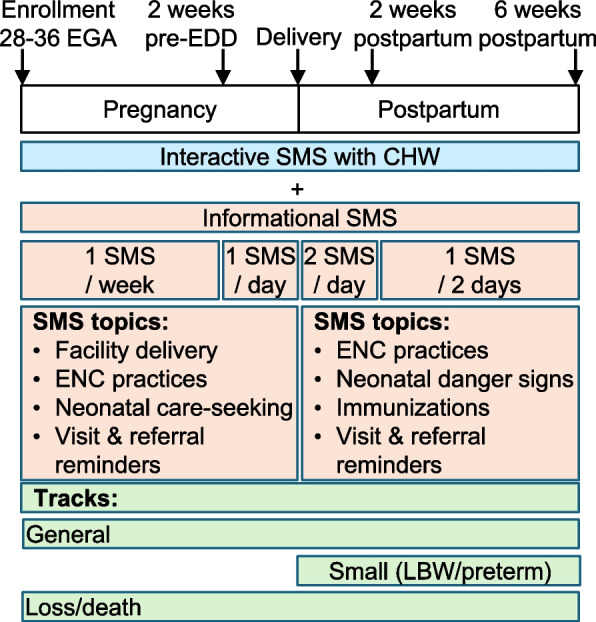
SMS curriculum overview

Automated SMS topics and schedule are summarized in Fig. 3, based on the client’s gestational age, date of delivery, and delivery outcome, obtained from eCHIS. From enrolment until 38 weeks gestation, automated SMS are sent weekly, encouraging facility delivery and birth planning, and providing anticipatory guidance about neonatal danger signs and emotional support. From 38 weeks gestation until delivery, automated SMS are sent daily, with similar content and highlighting neonatal danger signs and ENC practices, including immediate and exclusive breastfeeding, thermal, and cord care. From delivery to 2 weeks postpartum, two messages are sent per day: one message with screening questions regarding neonatal danger signs and encouragement to message if they have a concern and one message with educational content on postpartum care and complications, ENC practices, and emotional support. From 2 to 6 weeks postpartum, SMS are delivered every other day. Additionally, based on input from perinatal clients, CHWs, and policymakers in formative design activities, clients receive a reminder message when they are referred to the healthcare facility by the CHW, when they are due for their 6-week postpartum facility visit, and when a CHW is scheduled to conduct a home visit. All messages open with the participant’s name, state that the message is from their CHW, and contain a question to engage women in conversation. Clients who deliver a preterm or low birthweight baby receive a modified track of messages, which includes information about additional care for small and preterm babies, including Kangaroo Mother Care. Clients who experience a stillbirth or neonatal death receive a 4-week curriculum of weekly bereavement messages which provide condolences and emotional support; messages related to the infant are stopped. The message bank is available upon request.

Message content and schedule were developed by a group of content experts on the study team. Messages are based on the Mobile WACh NEO intervention [41], with the following modifications to account for the shift from nurse to CHW delivery and to respond to stakeholder input in our formative design activities. Terminology in the messages was simplified to remove technical clinical terms and better align with lay vocabulary used by CHWs. To align with CHWs’ preventative service focus, additional messages were added antenatally providing anticipatory guidance for ENC, nutrition counseling, and reminders about family planning. To align with CHWs’ referral responsibilities and limited diagnostic role, message language requesting participants describe symptoms of illness was removed and replaced with a recommendation to seek facility-based care in case of health concerns. Discussion of mental health topics was removed due to CHW input that they did not feel comfortable responding to in-depth messages about mental illness; instead, emotionally supportive language such as “I am here for you” was increased. Additionally, based on findings from the Mobile WACh NEO trial, the frequency of antenatal messages with anticipatory guidance related to trial outcomes, particularly infant danger signs, was increased, while the frequency of messages encouraging facility delivery was decreased, given the high rates of facility delivery currently in Kenya (> 80%) [43].

#### Integration of SMS into *eCHIS* application

**Fig. 4:**
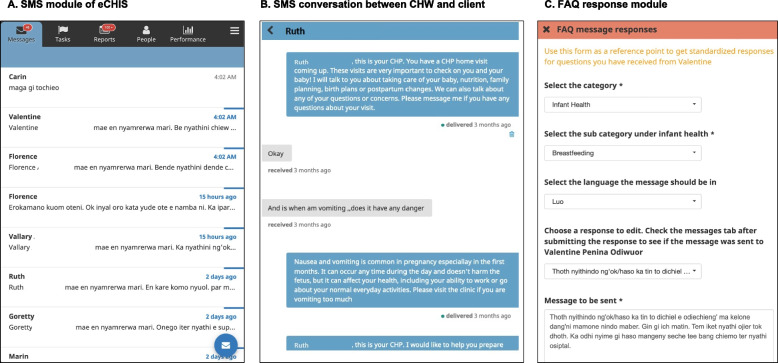
Screenshots of SMS components of eCHIS

In partnership with the Kenyan Ministry of Health, SMS messaging functionality was integrated into the eCHIS software platform as a module of the existing interface (Fig. 4). Consenting and enrollment to communicate by SMS with the CHW was added as a required step in the standard antenatal home visit workflow among intervention CHWs, and an additional functionality was added to update client phone number, SMS language, or messaging preferences after enrollment as needed. Templates for CHW replies to frequently asked client questions were incorporated as a “FAQ” module with a topically organized bank of editable message templates in English, Swahili, and Luo (Fig. 4).

In addition to integrating SMS functionality into CHW workflows, we incorporated supervision of SMS messaging into the workflows of CHW supervisors. As part of their monthly supervision workflow, supervisors are prompted to review a CHW’s messaging, ask them if they have experienced any challenges with messaging, and support them in problem-solving any challenges.

#### Training and launch activities

The study provided training to CHWs and supervisors at the study launch on the following topics. All CHWs and supervisors in control and intervention clusters received a 2.5-day training on how to use eCHIS. Those in intervention clusters received an additional day of training on how to use the CHV-NEO SMS workflows in eCHIS. To ensure a baseline level of clinical knowledge related to maternal and neonatal health, all CHWs in both arms also received a half-day refresher training on their clinical responsibilities related to antenatal and postnatal care, newborn health, and maternal mental health.

As part of training, CHWs were instructed to sync their eCHIS application with the server daily, respond to clients by SMS within 24 h, refer to the facility if any unusual symptoms are reported by SMS or if they were unsure how to respond, seek advice from their supervisors if they have challenges with any workflows, and report any technical issues to the study team.

### Criteria for discontinuing or modifying allocated interventions {11b}

#### SMS withdrawal

Clients in the intervention group may withdraw from receiving SMS messages at any time by either sending a SMS with the word “stop” or asking their CHW to stop their messaging. Participants who withdraw from SMS can continue or withdraw from study activities.

#### Study withdrawal

Participants may withdraw from the study at any time for any reason. Study staff record the timing and reason for any early terminations. All data collection will be discontinued at the time of withdrawal. Participants who withdraw from study activities can continue or withdraw from the SMS program.

### Strategies to improve adherence to interventions {11c}

CHWs in Kisumu County receive routine supervision to support their service delivery, in the form of monthly group meetings and individual meetings between CHWs and their supervisor. The study carries out the following additional quality assurance activities. At monthly group meetings, the study shares a report on aggregate CHW performance in terms of completeness of eCHIS data related to pregnancies and newborns and, at intervention facilities, SMS messaging process indicators. Additionally, the study team conducts a monthly review of a sample of CHWs’ SMS exchanges with clients. Clinical experts on the study team review CHW message timeliness and appropriateness per standards set forth in prior Mobile WACh studies [37, 41]. Summary data from these reviews and feedback for CHW responses are shared with CHWs and their supervisors at the monthly supervision meetings. The same data is also shared with Kisumu County leadership at separate regular meetings.

### Relevant concomitant care permitted or prohibited during the trial {11d}

Perinatal women participants in both arms continue to receive all standard healthcare services from local healthcare facilities. The study provides no clinical care.

### Provisions for post-trial care {30}

Participants continue with services as usual post-trial.

### Outcomes {12}

#### Clinical outcomes

Primary, secondary, and exploratory clinical outcomes are summarized in Table 1. The primary outcome is neonatal mortality, defined as the death of a live-born infant within 28 days of birth, based on routine data abstracted from eCHIS during the study period. Secondary outcomes are ascertained among the in-depth perinatal cohort who complete study questionnaires. Secondary outcomes were selected based on the intervention’s hypothesized mechanism of action (Fig. 2) and their importance as intermediate outcomes in neonatal survival. These include initiation of breastfeeding within the first hour of life (ascertained by maternal self-report at the 6-week postpartum study visit); application of substances to cord (ascertained by maternal self-report at the 6-week visit); maternal knowledge of neonatal danger signs (defined as the number of the eight neonatal danger signs correctly named at the 6-week visit); and appropriate care seeking (defined as the proportion of illness episodes with danger signs where the infant attended the clinic, based on maternal self-report at the 6-week visit). Exploratory clinical outcomes include mothers’ identification of danger signs in their infants (defined as the proportion of all infants whose mothers report they ever exhibited a danger sign at the 6-week visit); infant hospital admissions (based on maternal self-report); and neonatal care delays (defined as the proportion of neonatal deaths attributable to each delay in the three delays model, based on review of adverse event reports by nurses and physicians on the study team).

**Table 1:** Trial outcomes

Outcome	Indicator	Source and timing	Statistical analysis
Primary clinical outcome			
Neonatal mortality	Death during the first 28 days of life	eCHIS routine data throughout 2 years of study implementation	GLMM (RE by facility and CHW) with Poisson link and survival time as offset
Secondary clinical outcomes			
Initiation of early breastfeeding	Breastfed in the first hour of life	Questionnaire at 6-week visit	GLMM (RE by facility and CHW) with Poisson link and robust SE
Appropriate cord care	Application of nothing or chlorhexidine to umbilical cord	Questionnaire at 6-week visit	GLMM (RE by facility and CHW) with Poisson link and robust SE
Appropriate thermal care	No bath in the first 24 h of life	Questionnaire at 6-week visit	GLMM (RE by facility and CHW) with Poisson link and robust SE
Maternal knowledge of neonatal danger signs	Number of the eight danger signs or symptoms successfully named	Questionnaire at 6-week visit	GLMM (RE by facility and CHW) with Poisson link
Appropriate care-seeking	Proportion of illness episodes with danger signs where the infant attended the clinic	Questionnaire at 6-week visit; adverse event monitoring throughout follow-up	GLMM (RE by facility and CHW) with Poisson link and robust SE
Exploratory clinical outcomes			
Identification of danger signs	Proportion of all infants whose mothers report history of ≥ 1 neonatal danger sign at any time	Questionnaire at 6-week visit	GLMM (RE by facility and CHW) with Poisson link and robust SE
Neonatal care delays	Proportion of neonatal deaths attributable to delays in care-seeking vs. other delays	Clinician review and classification of adverse event reports	Chi-square test
Appropriate care-seeking	Number of hospital admissions during the first 6 weeks of life	Questionnaire at 6-week visit; adverse event monitoring throughout follow-up	GLMM (RE by client, facility and CHW) with Poisson link and robust SE
Exploratory service delivery and implementation outcomes			
Home visit coverage	Number of home visits performed per CHW per month	eCHIS routine data throughout 2 years of study implementation	GLMM (RE by facility and CHW) with Poisson link
Perinatal clinic referral	Number of clinic referrals logged in eCHIS for clients who are pregnant or up to 3 months postpartum per month	eCHIS routine data throughout 2 years of study implementation	GLMM (RE by facility and CHW) with Poisson link
CHW workload	Time on all CHW duties	Questionnaire at 1 year and study close	GLMM (RE by facility and CHW) with identity link
Supervisor workload	Time on all supervisor duties per week	Questionnaire at 1 year and study close	GLMM (RE by facility) with identity link
CHW acceptability	AIM score	Questionnaire at 1 year and study close	Not compared between arms
Perinatal client acceptability	AIM score	Questionnaire at 6-week visit	Not compared between arms
CHW adoption	Proportion of client messages replied to per CHW	eCHIS routine data throughout 2 years of study implementation	Not compared between arms
CHW fidelity	Proportion of client messages replied to within 24 h per CHW	eCHIS routine data throughout 2 years of study implementation	Not compared between arms
CHW fidelity	Proportion of client messages replied to in an appropriate manner per CHW	Clinician review of eCHIS routine data throughout 2 years of study implementation	Not compared between arms

*GLMM* generalized linear mixed models, *RE* random effects, *SE* standard error

#### Service delivery and implementation outcomes

In addition to clinical outcomes, we will compare service delivery outcomes between arms as exploratory outcomes (Table 1). These outcomes are ascertained from eCHIS data and self-report questionnaires from all CHWs (*N* = 700). These include home visit coverage (defined as number of home visits performed by CHWs); perinatal clinic referrals (defined as number of clinic referrals logged by CHWs in eCHIS for clients who are pregnant or up to 3 months postpartum); and CHW and supervisor workload (defined as time on all work duties, based on self-report questionnaire).

Within the intervention arm, CHV-NEO acceptability, adoption and fidelity of use by CHWs and their supervisors is determined using questionnaires and eCHIS data. Acceptability is defined as perception by CHWs that CHV-NEO is agreeable or satisfactory, using the AIM scale in CHW self-report questionnaire [48]; adoption is defined as the proportion of client SMS replied to by CHWs at all based on eCHIS data; and fidelity is defined as the proportion of client SMS responded to by CHWs in an appropriate and timely fashion, ascertained through monthly structured review and scoring of a sample of SMS conversations by clinically trained study team members. Quantitative data from CHW and supervisor questionnaires as well as qualitative interviews with CHWs and supervisors are used to define CHW-, supervisor-, and facility-level drivers of acceptability, adoption, and fidelity.

### Participant timeline {13}

Peripartum women participants at control and intervention facilities complete questionnaires at enrollment into the study and at 6 weeks postpartum (Table 2). CHWs and supervisor participants complete questionnaires at study launch, 1 year after launch, and study close. CHW and supervisor qualitative interviews are conducted 1 year after launch and at study close.

**Table 2:** CHV-NEO RCT participant schedule

Timepoint	Cluster randomization	Intervention deployment	Post-intervention deployment		RCT mid-point	RCT close-out
	***-t***_***2***_	***-t***_***1***_	**0**	***t***_***1***_	***t***_***2***_	***t***_***3***_
Peripartum women			Enrollment visit	6-week postpartum follow-up visit		
Allocation	*X*					
Eligibility screen			*X*			
Baseline ascertainment			*X*			
Clinical outcome ascertainment				*X*		
Closeout procedures				*X*		
CHWs and supervisors		Enrollment visit			1 year visit	Study close visit
Allocation	X					
Eligibility screen		X				
Informed consent		X				
Baseline ascertainment		X				
Mid-point qualitative and quantitative					X	
End-point qualitative and quantitative						X

Timepoints *-t*_*2,*_* -t*_*1,*_* t*_*2,*_* t*_*3*_ are on the trial implementation timeline, whereas timepoints 0 and *t*_*1*_ are on each participant’s pregnancy timeline

### Sample size {14}

Our study is powered for primary and secondary clinical outcomes. The cluster-randomized trial includes 20 facility clusters, 10 interventions, and 10 controls. For ascertainment of our primary outcome of neonatal mortality, we will abstract outcomes from eCHIS on 7200 pregnancies ending in live births across the clusters. With this sample size, assuming *α* = 0.05, two-sided tests, 10% attrition, a conservative coefficient of variation of 0.25, and assuming unequal cluster sizes with an average cluster size 360, we have 80% power to detect a difference in neonatal mortality of 21.0 vs. 10.3 per 1000 live births [43]. For ascertainment of secondary outcomes, we will enroll 2000 pregnant clients in the in-depth perinatal cohort. With this sample size, assuming *α* = 0.05, two-sided tests, 10% attrition, coefficient of variation of 0.25, and unequal cluster sizes (ranging between 80 and 120 per cluster), we have 80% power to detect a difference in early initiation of breastfeeding, thermal care, and cord care of 50% vs. 72%. We expect the outcomes of appropriate care-seeking and knowledge of danger signs to be correlated so we will correct these analyses for multiple comparisons. Assuming a Bonferroni-adjusted *α* = 0.025, we will have 80% power to detect a difference between arms of 2.0 vs. 2.4 danger signs named and 0.5 vs. 0.9 appropriate clinic visits attended in the first 6 weeks [6, 37, 49, 50].

Service delivery and implementation outcomes are ascertained among the in-depth perinatal cohort as well as all CHWs and supervisors providing services over the 2-year period (estimated sample size 700 CHWs and 40 supervisors). Quantitative and qualitative analyses of implementation outcomes are intended to be exploratory; no formal hypothesis testing will be performed. We expect sample sizes for qualitative interviews will be sufficient to reach theoretical saturation of themes [51].

### Recruitment {15}

#### Abstracted mortality cohort

Mortality is ascertained from the abstracted data from all perinatal clients enrolled in eCHIS in the study areas meeting the inclusion criteria. Clients are registered in eCHIS through CHWs’ routine home visit activities.

#### In-depth perinatal cohort

Participants in the in-depth perinatal cohort are recruited during ANC visits at the 20 randomized study facilities. Potential participants are approached by MCH facility staff and referred to the study staff at each clinic to obtain additional information. Study staff emphasize that participation is completely voluntary and will not affect their access to ANC, postnatal, or infant care services.

#### CHWs and supervisors

CHWs and supervisors at all study facilities are recruited by study staff for participation in questionnaires through facility-wide group meetings or announcements from facility leadership at study facilities. CHWs or supervisors who join the study facility part-way through the study are recruited to complete subsequent rounds of data collection (1 year or trial close). It is emphasized that participation is completely voluntary and would not in any way affect their employment.

A subset of 50 CHWs and 10 supervisors at intervention sites are selected for individual interviews 1 year after the study trial launch and at trial close. Interview participants are selected based on exhibiting below-median vs. above-median fidelity to contrast the experiences of the two groups and identify constructs associated with variations in implementation. Selected individuals are contacted through facility leadership and invited to contact the study to learn more about participation. They and facility leadership are not be told why they were selected, to avoid any negative consequences from their workplace.

### Assignment of interventions: allocation

#### Sequence generation {16a}, concealment mechanism {16b}, and implementation {16c}

Facility-level cluster randomization was conducted using a restricted randomization approach. Facilities were stratified into eight groups of two to six facilities, based on similar characteristics that may influence outcomes of interest, including facility level, patient volume, rural/urban designation, and services offered. Randomization was conducted through an interactive event using an online visual simulation of randomization [52]. A representative from each facility “spun” an online “wheel” to determine their order of assignment within stratified groups and a second wheel to determine final assignment within the randomization stratum.

### Assignment of interventions: blinding

#### Who will be blinded {17a}

This will be a non-blinded study. All facilities, trial participants, and analysts will have access to intervention assignment information. Study co-investigators will only review outcome data in aggregate across arms during the trial.

#### Procedure for unblinding if needed {17b}

N/a. This study is unblinded.

### Data collection and management

#### Plans for assessment and collection of outcomes {18a}

Data are collected through three primary modes: abstraction of eCHIS data, quantitative questionnaires with perinatal women, CHWs and supervisors, and qualitative interviews with CHWs and supervisors.

#### *eCHIS* data abstraction

eCHIS data, including task completion and SMS messaging, is extracted directly from the eCHIS server.

#### Quantitative questionnaires

Quantitative questionnaires are administered electronically to women, CHWs, and supervisors by study staff using the REDCap platform. Women complete questionnaires during study visits at the facility at enrollment in pregnancy and at 6 weeks postpartum (Table 2). At each visit, a standardized questionnaire is administered to record self-reported outcomes and sociodemographic and clinical characteristics that may be associated with them. Outcomes include timing of breastfeeding initiation, introduction of complementary foods, application of substances to the umbilical cord, knowledge of eight danger signs (not feeding, high temperature, low temperature, fast breathing, difficulty breathing, not moving, convulsions, and jaundice) [53], timing of first bath, attending facility-based care during illness episodes, and acceptability of CHV-NEO, using the AIM [48]. Associated demographic and clinical factors include maternal education, income, employment, parity, distance from home to clinic, intimate partner violence, depression symptoms, past healthcare experience, parental self-efficacy, and infant sex.

CHWs and supervisors complete questionnaires annually: at study launch, 1 year, and study close (Table 2). Questionnaires include outcomes as well as demographic and professional characteristics that may be associated with outcomes. Outcomes include acceptability of CHV-NEO, assessed using the AIM measure [48], and time spent on work duties in the prior 2 weeks. Characteristics that may be associated with outcomes include professional background, training, and experience and comfort with the eCHIS platform.

#### Qualitative interviews

We conduct individual semi-structured interviews with a sample of CHWs and supervisors at intervention sites 1 year after the study trial launch and at the trial close. Interview guides explore determinants of implementation success, comparing CHWs exhibiting low vs. high fidelity delivery of the intervention. Discussion guides are based on constructs from the consolidated framework for implementation research (CFIR), adapted for use in LMIC settings [54]. IDIs are conducted by a trained social scientist fluent in English, Swahili, and Luo and audio-recorded, transcribed verbatim, and translated into English.

#### Plans to promote participant retention and complete follow-up {18b}

A study staff member is stationed at each facility and is responsible for the recruitment and follow-up of participants. Participants who are 2 weeks late for their 6-week study visit are actively traced by phone call and home visit to maximize completeness of 6-week visit data. We successfully used this approach in previous RCTs [55]. Data from participants who discontinue the study will be included in the analysis up to the point of discontinuation.

#### Data management {19}

Abstracted data is collected through direct extraction from eCHIS via a secure web-based login. Data is extracted and stored on a secure University of Washington server with restricted access. Questionnaire data is collected using the REDCap online version. Personal identifying information and consent forms are stored in a separate REDCap project from study data. All REDCap projects require login and password information unique to individual study staff. Data collectors receive study-specific training on all questionnaires and surveys. A data manager reviews all new enrollments and survey entries daily using quality control reports within REDCap and confirms end-of-day enrollments and questionnaire numbers with data collectors.

#### Confidentiality {27}

Personal identifying information is only collected for the in-depth cohort, CHWs, and supervisors. Personal information is stored separately from study data. Data abstracted from eCHIS does not include client identifiers such as names or phone numbers. Data is only collected and accessed by study staff who have received human subjects and data protection training through a secure server.

#### Plans for collection, laboratory evaluation, and storage of biological specimens for genetic or molecular analysis in this trial/future use {33}

N/a. This study will not be collecting any biological specimens.

### Statistical methods

#### Statistical methods for primary and secondary outcomes {20a}

Statistical analysis approaches for each outcome are summarized in Table 1. Analysis of primary and secondary outcomes will be by intention-to-treat. If participants relocate between facilities in different intervention arms during the course of the study, the original assignment is used in the analysis. For analyses that include random effects at the CHW level, if a participant’s assigned CHW changes, their data is analyzed under the CHW under whose care they are for the longest proportion of follow-up time preceding outcome ascertainment. Analyses will be adjusted for any baseline characteristics that differ significantly between study arms.

#### Interim analyses {21b}

An interim analysis for neonatal mortality will be performed using O’Brien-Fleming boundaries for benefit and harm when 50% of expected person time has been accrued. The study’s data safety monitoring board (DSMB) will meet to review the interim analysis and may conclude with recommendations to continue the trial without change, modify the trial, or terminate the trial.

#### Methods for additional analyses (e.g., subgroup analyses) {20b}

We have no pre-specified subgroup analyses.

#### Methods in the analysis to handle protocol non-adherence and any statistical methods to handle missing data {20c}

Based on the Mobile WACh NEO RCT [41], we expect a 10% loss to follow-up between enrollment and the 6-week study visit. We anticipate that women who are lost to follow-up are more likely to be disengaged from care and have experienced adverse outcomes. We also anticipate missingness may differ by arm, with the intervention arm having higher retention. A secondary analysis of mortality will be performed in which missing mortality at 28 days will be imputed using multiple imputations by chained equations (MICE).

To explore any dose–response effects associated with variation in the fidelity of intervention delivery, we will conduct an exploratory analysis comparing primary and secondary outcomes between participants who received low vs. high fidelity of the intervention, defined based on the number of automated and CHW messages received. Propensity score matching will be used to control for confounding by participant or CHW characteristics.

#### Plans to give access to the full protocol, participant-level data, and statistical code {31c}

De-identified data, statistical code, and the study protocol will be made available to the public upon publication of the results.

### Oversight and monitoring

#### Composition of the coordinating center and trial steering committee {5d}

The University of Washington, Kenyatta National Hospital, and Medic teams all contribute to trial organization and daily support. Day-to-day trial operations are undertaken by data collectors, a data manager, and a research coordinator based in Kisumu County. This team meets with the rest of the team (research coordinator, research scientists, design specialists, co-investigators, and principal investigators) weekly to report on progress and review data.

#### Composition of the data monitoring committee, its role, and reporting structure {21a}

The CHV-NEO study has engaged an independent Data Safety Monitoring Board (DSMB) to act in an advisory capacity to monitor patient safety and evaluate the efficacy of the intervention. The responsibility of the DSMB is to review interim safety and efficacy at periodic intervals (6-monthly intervals after initiation of the trial). The Data Safety Monitoring Board consists of physicians, statisticians, neonatologists, and digital health specialists selected by the study team. Membership consists of persons completely independent of the investigators who have no financial, scientific, or other conflict of interest with the trial. Members are not current or past collaborators or associates of the University of Washington or Kenyatta National Hospital principal investigators.

#### Adverse event reporting and harms {22}

Adverse events among perinatal women, CHWs, or supervisor participants are elicited at all study visits and may also be reported spontaneously if a participant contacts the team. All adverse events are recorded by study staff using an electronic REDCap questionnaire based on a phone conversation with the participant. Events to be monitored include maternal and infant death, maternal and infant hospitalization, pregnancy loss, experience of violence, losing housing, breach of confidentiality, and suicidal behavior. Adverse event monitoring in the mortality cohort is more limited because the study will not have direct contact with these individuals. Events monitored in this cohort include maternal and infant death, pregnancy loss, and report of maternal postpartum danger signs at home visits, abstracted from eCHIS. Adverse and severe adverse events will be monitored by the study team, reported to ethical review boards as needed, and unblinded results will be reviewed by the DSMB. The DSMB will make recommendations regarding any imbalances in safety outcomes.

#### Frequency and plans for auditing trial conduct {23}

No audits are planned.

#### Plans for communicating important protocol amendments to relevant parties (e.g., trial participants, ethical committees) {25}

Modifications and amendments to study protocols are submitted to the University of Washington Institutional Review Board and Kenyatta National Hospital Ethics Review Committee before implementation. Once approved, participants are informed and re-consented at scheduled study visits, as needed, to align with updated protocols. Changes are communicated to the DSMB at regularly scheduled meetings. Trial registries are reviewed and updated by the study team yearly during the study period.

#### Dissemination plans {31a}

We will disseminate results to a community advisory board, made up of medical providers and community members living in the study area, twice a year. Study findings will be shared with the participating facilities, Kisumu County leadership, and Kenyan Ministry of Health at the study conclusion as a presentation or written report. Findings will be disseminated to the research community in the form of conference presentations and journal articles.

## Discussion

This trial will be the first to evaluate the effect of a text messaging intervention provided by CHWs on neonatal health. Integration of our intervention into the Ministry of Health’s eCHIS, provision of the intervention by County CHWs as part of their routine services, and evaluation of implementation outcomes will ensure that findings from this trial are relevant to routine care provision and readily translated to policy decisions. Findings from this trial have the potential to inform the design and implementation of future text messaging programs with CHWs in LMICs.

## Trial status

We began recruitment on December 11, 2023, and anticipate the completion of data collection in December 2025. The current protocol is version 5, dated 28 November 2023.

## Data Availability

Deidentified data will be made available upon publication of trial findings, in compliance with a data sharing agreement between Kisumu County and the University of Washington.

## Abbreviations

ANC: Antenatal care
eCHIS: Electronic Community Health Information System
ERC: Ethics Review Committee
CHW: Community health worker
IDI: In-depth interview
IRB: Institutional Review Board
LMIC: Low- and middle-income country
MCH: Maternal and child health
MOH: Ministry of Health
RCT: Randomized controlled trial
SMS: Short message service
SOP: Standard operating procedure
WHO: World Health Organization
DSMB: Data Safety Monitoring Board
